# Supplementary material for: MetaRibo-Seq measures translation in microbiomes
Source: Nat Commun. 2020 Jun 29;11:3268. doi: 10.1038/s41467-020-17081-z (PMC7324362; doi:10.1038/s41467-020-17081-z)
Supplement: Supplementary file 10 — Supplementary Data 7 [file 41467_2020_17081_MOESM10_ESM.zip › File2/Confidence_High_Taxonomy/320892_out.krona.html]

Javascript must be enabled to view this page.

members
magnitude
magnitudeUnassigned
count
unassigned
taxon
rank

320892\_out

4

2
superkingdom
4

4
1239
phylum

4
186801
class

order
186802
4

4
family
541000

1
1898205

SRS1041031\_contig\_number\_contig-100\_27503.27504
species

1263
genus
3

1262959

SRS078176\_contig\_number\_3483
species
1

2

SRS013687\_contig\_number\_contig-100\_4580.95480SRS147557\_contig\_number\_27377
species
1262961
